# Sunlight-Induced photochemical synthesis of Au nanodots on α-Fe_2_O_3_@Reduced graphene oxide nanocomposite and their enhanced heterogeneous catalytic properties

**DOI:** 10.1038/s41598-018-24066-y

**Published:** 2018-04-09

**Authors:** G. Bharath, Shoaib Anwer, R. V. Mangalaraja, Emad Alhseinat, Fawzi Banat, N. Ponpandian

**Affiliations:** 1 Department of Chemical Engineering, Khalifa University of Science and Technology, P.O. Box 127788, Abu Dhabi, United Arab Emirates; 2 Advanced Ceramics and Nanotechnology Laboratory, Department of Materials Engineering, Faculty of Engineering, University of Concepcion, Concepcion, Chile; 3 Department of Mechanical Engineering, Khalifa University of Science and Technology, P.O. Box 127788, Abu Dhabi, United Arab Emirates; 4 Department of Chemical Engineering, Petroleum Institute, Khalifa University of Science and Technology, Abu Dhabi, United Arab Emirates; 5 Department of Nanoscience and Technology, Bharathiar University, Coimbatore, Tamilnadu India

## Abstract

In this present study, we report the synthesis of Au nanodots on α-Fe_2_O_3_@reduced graphene oxide (RGO) based hetero-photocatalytic nanohybrids through a chlorophyll mediated photochemical synthesis. In this process, chlorophyll induces a rapid reduction (30 min) of Au^3+^ ions to Au° metallic nanodots on α-Fe_2_O_3_@RGO surface under sunlight irradiation. The nucleation growth process, photo-induced electron-transfer mechanism and physico-chemical properties of the Au@α-Fe_2_O_3_@RGO ternary nanocomposites were systematically studied with various analytical techniques. This novel photochemical synthesis process is a cost-effective, convenient, surfactant-less, and scalable method. Moreover, the prepared ternary nanocomposites enhanced catalytic activity as compared to pure α-Fe_2_O_3_ and α-Fe_2_O_3_@RGO. The advantages and synergistic effect of Au@α-Fe_2_O_3_@RGO exhibit, (i) a broader range of visible-light absorption due to visible light band gap of α-Fe_2_O_3_, (ii) lower recombination possibility of photo-generated electrons and holes due to effect of Au and (iii) faster electron transfer due to higher conductivity of RGO. Therefore, the prepared Au@α-Fe_2_O_3_@RGO hetero-photocatalytic nanohybrids exhibited a remarkable photocatalytic activity, thus enabling potential active hetero-photocatalyst for industrial and environmental applications.

## Introduction

Recently, nanohybrids hetero-nanostructures integrating two-dimensional (2D) graphene with metal oxides have received increased consideration in recent years^1–8^. Specifically, graphene and hetero-photocatalysts have been broadly used for high-performance photocatalysis, which is a highly important for energy conversion devices. Especially, photocatalytic degradation and water splitting has attracted considerable research interest and made encouraging progress, in which inorganic nanostructures such as, MoS_2_, TiO_2_, CdS, Mn doped α-Fe_2_O_3_, α-Fe_2_O_3_/3D graphene, graphene oxide, GO/SiC, α-Fe_2_O_3_/reduced graphene oxide and Au-supported CeO_2_^9–16^. Among them, α-Fe_2_O_3_ nanoparticles (NPs) is a promising visible light photocatalysts because of its very low cost, chemically stable and favorable band gap (2.1–2.2 eV) to absorb photons in the visible light range. However, its photocatalytic efficiency is delayed by the short lifetime of the photo generated charge carriers (<10 ps), short hole diffusion length (2–4 nm), and poor mobility of charge carriers (<0.2 cm^2^·V^−1^·s^−1^). Conversely, α-Fe_2_O_3_ NPs still suffers from a high charge recombination rate because of boundaries between the nanoparticles. Resent Massive efforts are carried out trying to improve the charge separation issue by providing a more mobile hole pathway^17,18^. Specifically, incorporating α-Fe_2_O_3_ NPs with nanostructured carbon materials (such as fullerenes, carbon nanotubes, and reduced graphene oxide (RGO) sheets) has unique advantages in suppressing the charge recombination. The RGO nanosheets are particularly effective in separating charges on α-Fe_2_O_3_ because of their 2D profile. Graphene, a unique 2-Dimensional monolayer of sp2- bonded carbon atoms (C=C) with π electron cloud in a closely packed honeycomb structured material, has received important consideration for photocatalytic and energy production applications. Owing to its extraordinary properties, such as large specific surface area, excellent electrical and thermal conductivity, good light transmission and high mechanical strength, graphene-based materials have been developed for active materials of energy storage and conversion, transparent conductors, nanoelectronics, and chemical sensors, *etc*.^19–22^.

Many recent articles have been contributed to improving the photocatalytic performance of Cu-Cu_2_O-graphene, TiO_2_/graphene, α-Fe_2_O_3_/RGO, CdS/RGO and SiC/RGO nanocomposites^15,16,22–24^. The incorporation of graphene or RGO with metal oxide can enhance the photocatalytic activity. It is claimed that photocatalysis enhancement by graphene is due to the fact that graphene provides a pathway for transport of charge carriers. Further, we increase the photocatalytic efficiency to incorporate the co-catalyst like noble metals (Au, Pd, and Pt) on metal oxide/graphene nanocomposites. In this context, a few metallic nanocomposites, such as, polymer protected Pt/Ru bimetallic clusters, Au/Pd core-shell nanoparticles, bimetallic Au/Pt systems, Au/TiO_2_, have been investigated for photocatalytic H_2_ production through photo-induced water splitting^24,25^. These results show that the hetero-catalysts deliver good performances in hydrogenation reactions as a result of longer electron mobility as well as surface ensemble effects. In addition, the adaptable properties of Au nanoparticles to alter the physicochemical properties, surface plasmon resonance (SPR), conductivity, and redox behavior, have proved Au to be a suitable nanomaterial leading to improvement in catalytic properties. Also supporting of gold nanoparticles is possible to introduce visible-light response in the α-Fe_2_O_3_/RGO that otherwise would be inactive under visible-light irradiation. The incorporating of Au species helps as a promoter to improve the catalytic performance of degradation of dyes and water splitting by modifying the route of photoelectron delivery.

Trending research focus is to develop facile and green reduction approaches with tailor control of size and shape of Au/α-Fe_2_O_3_/graphene nanohybrids^26^. An interesting green chemistry approach of interest is the photochemical synthesis of metallic nanoparticles under UV-light exposure. In general, Au NPs are prepared in the presence of ethylene glycol and poly(vinylpyrrolidone) (PVP) through photochemical reduction of Au^3+^ ions^16^. Huang *et al*.^20^, demonstrated photochemical reduction and shape-controlled fabrication of anisotropic Au nanostructures on semiconducting nanostructures of TiO_2_ sols and highly crystalline oriented ZnO NPs arrays^27^. Notably, Au nanorods were grown on graphene oxide (GO) hybrids by one-pot photochemical process via UV light irradiation, where Au nanorods are formed on the surface of GO sheets. However, the photochemical methods developed for synthesis of Au@graphene and Au@metal oxide (TiO_2_, ZnO) nanohybrids do not result in hetero-nanostructures with controllable shape, size and morphology^28^. Therefore credence in an improved green photochemical reduction process for rapid synthesis of size controlled Au nanodots on graphene/metal oxide ternary nanocomposites, especially towards enhancing photocatalytic performance.

To this end, we have developed an innovative environmentally friendly photochemical route for synthesis of Au nanodots on α-Fe_2_O_3_/RGO ternary nanocomposites. We report a highly crystalline α-Fe_2_O_3_/RGO which was synthesized through hydrothermal method at 180 °C for 12 h. The catalytic sites of α-Fe_2_O_3_ were encapsulated within the RGO sheets, which prevents the aggregation of individual nanoparticles and agglomeration of RGO sheets. The resulting sheets maintain their high specific surface area to enhance electrocatalytic behavior. Additionally, a novel photochemical reduction of Au^3+^ on α-Fe_2_O_3_@RGO hetero-nanostructure under sun light irradiation with presence and absence of chlorophyll was demonstrated. The physico-chemical properties of the ternary nanocomposites were investigated through varieties of analytical tools such as X-ray diffraction (XRD), Fourier transform infrared (FTIR), Raman spectroscopy, UV-Vis spectroscopy, Field emission scanning electron microscopy, High resolution transmission electron microscopy (HRTEM), and X-ray photoelectron spectroscopy (XPS). The photo chemically synthesized Au@α-Fe_2_O_3_@RGO nanocomposites exhibit the enhanced photocatalytic activity toward the degradation of methylene blue under sunlight irradiation. The mechanism of photocatalytic dye degradation was proposed for the degradation of methylene blue is illustrated in the present investigation. We hope that this present investigation can provide as a basis for further design of Au@α-Fe_2_O_3_@RGO nanocomposites catalyst for water purification and photochemical water splitting applications.

## Results and Discussion

### Characterization results of α-Fe_2_O_3_@RGO and Au@α-Fe_2_O_3_@RGO nanocomposites

**Figure 1:**
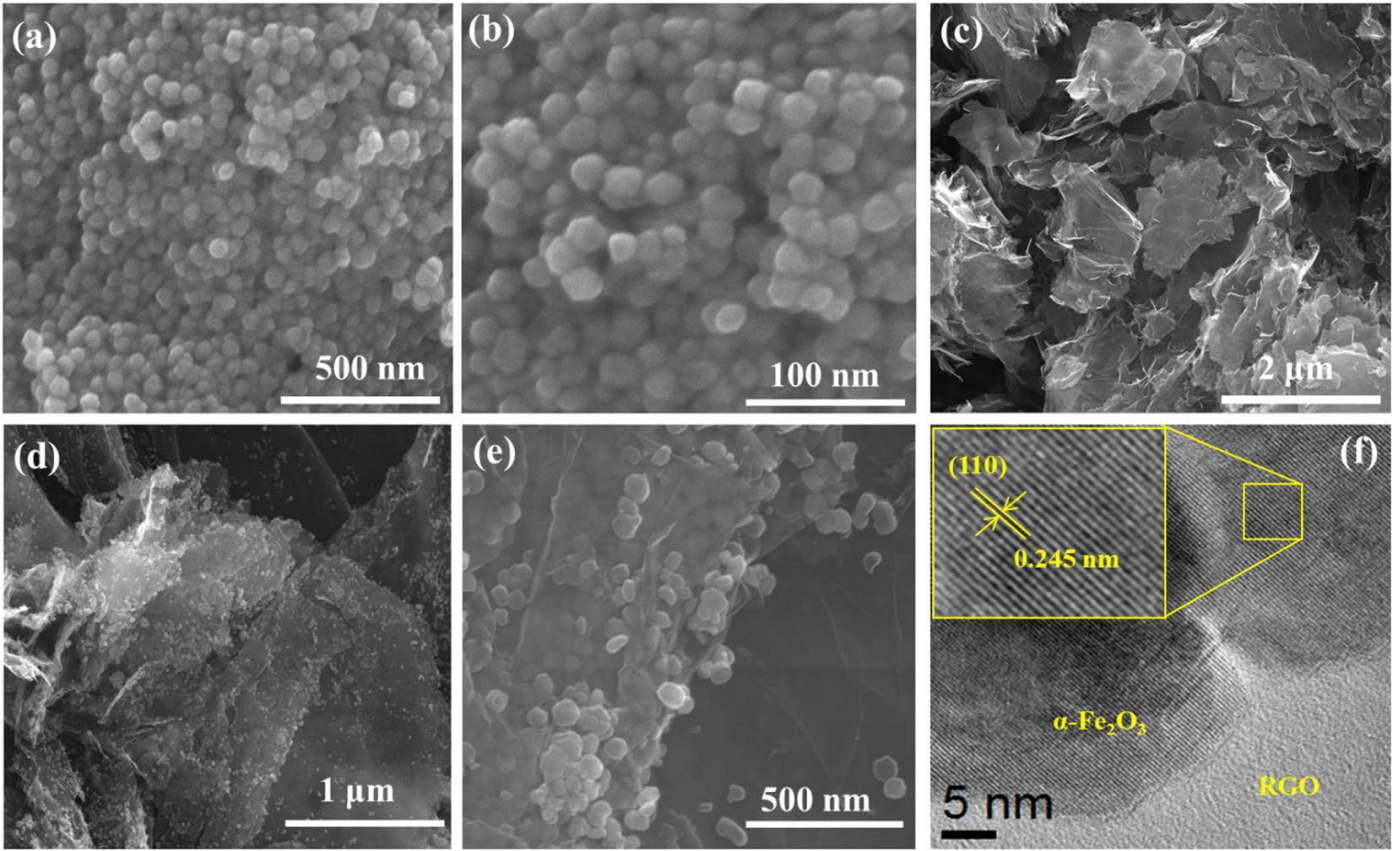
(**a**) and (**b**) low and high magnification FESEM images of α-Fe_2_O_3_ nanoparticles, (**c**) FESEM image of RGO, (**d**) and (**e**) low and high magnification FESEM images ofα-Fe_2_O_3_@RGO and (**f**) HRTEM image of a single α-Fe_2_O_3_ NPs on RGO sheets with its corresponding lattice line pattern.

The hydrothermally and photochemically synthesized samples were characterized with different analytical tools to understand their physico-chemical properties. The FESEM and HRTEM analysis were used for characterization of size, morphology and crystallinity of α-Fe_2_O_3_, RGO and α-Fe_2_O_3_@RGO nanocomposites as shown in Fig. 1(a–f). Low and high magnification FESEM images of prepared α-Fe_2_O_3_ reveal that the particles were monodisperse sphere-like particle with a narrow size distribution. The typical low-magnification FESEM image (Fig. 1(a)) visibly displays that the α-Fe_2_O_3_ possesses uniform size. The high magnification FESEM image shown in Fig. 1(b) indicates that the obtained nanoparticles were ~42 nm in diameter with sphere-like morphology, consistent with that evaluated from Scherrer’s formula. The morphology of the RGO sheets prepared via hydrothermal reduction process was observed through FESEM as shown in Fig. 1(c). It reveals that the RGO sheet consists of randomly well dispersed and thin sheets with characteristic folds on the surface. This result supported that the GO was successfully reduced to two dimensional nanosheets of RGO by hydrothermal reduction at 180 °C, alleviating the need for use of toxic chemical reducing agents^18^. Further, the morphology of the crystalline structure of the α-Fe_2_O_3_@RGO was elucidated through FESEM and HRTEM. The low-magnification FESEM image of α-Fe_2_O_3_@RGO nanocomposites in Fig. 1(d) showed a thin and wrinkled like RGO nanosheets with uniform dispersed α-Fe_2_O_3_ nanoparticles. The high-magnification FESEM image in Fig. 1(e) shows that the α-Fe_2_O_3_ nanoparticles were uniformly well dispersed on the surface of RGO sheets with average particle sizes of ~38 nm. Evidently, most of the α-Fe_2_O_3_ nanoparticles were encapsulated within the RGO sheets, which confirms the prevention of aggregation of individual nanoparticles and agglomeration of RGO sheets, thus maintaining a high specific surface area. The HRTEM image of the individual morphology of α-Fe_2_O_3_ nanoparticles on RGO nanocomposites are revealed in Fig. 1(f) and the inset HRTEM image in Fig. 1(f) shows the clear shell lattice fringe of α-Fe_2_O_3_ with d-spacing of 0.245 nm, corresponding to the (110) plane of hexagonal α-Fe_2_O_3_.

**Figure 2:**
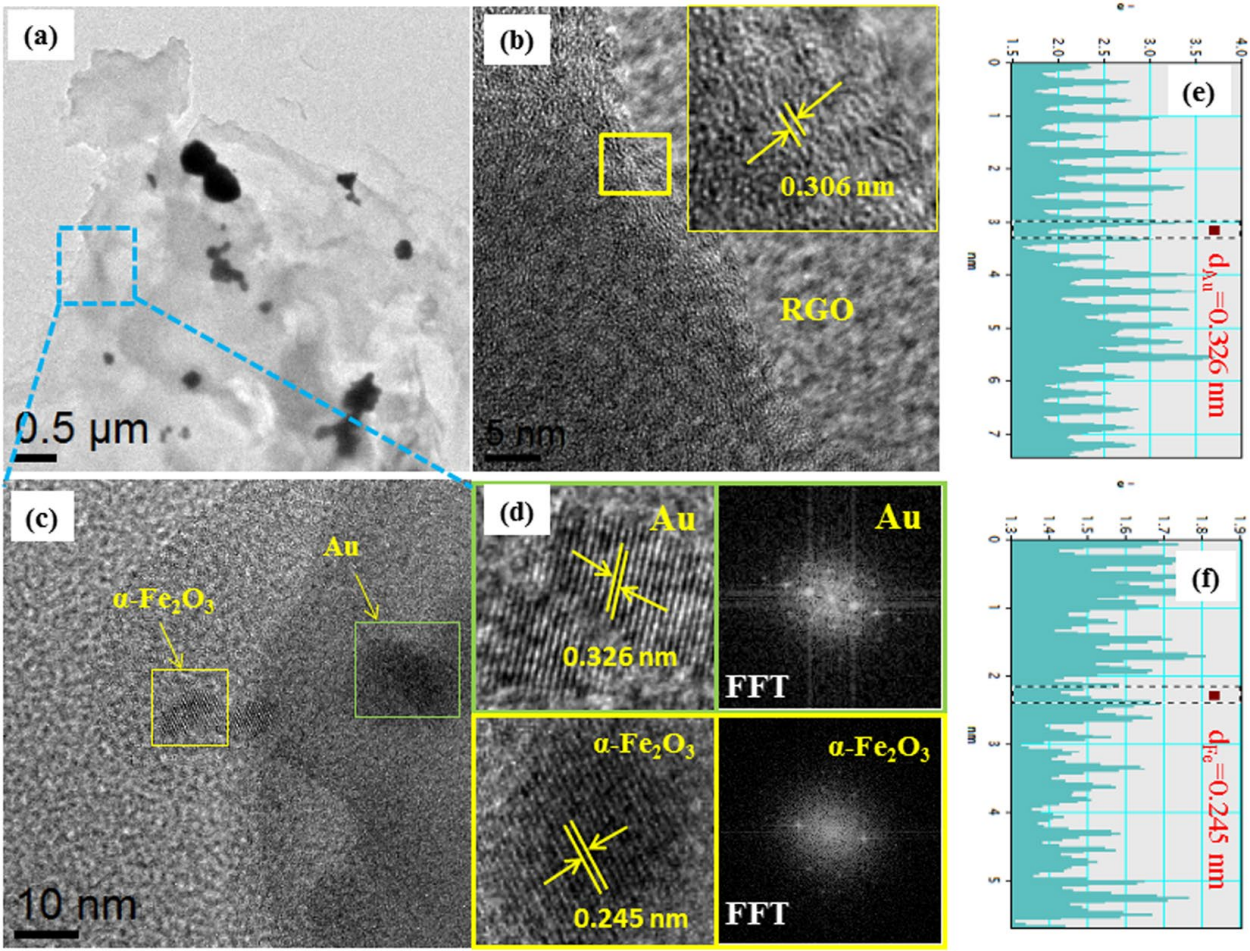
HRTEM images, lattice line and FFT pattern of Au@α-Fe_2_O_3_@RGO ternary nanocomposites and the statistical line profile analysis of HRTEM images showing lattice spacing’s of 0.326 and 0.245 nm, corresponds to Au and α-Fe_2_O_3_, respectively in the Au@α-Fe_2_O_3_@RGO ternary nanocomposites.

Further, morphology of the photochemically Au@α-Fe_2_O_3_@RGO ternary nanocomposites were examined by HRTEM and crystallinity of the samples were confirmed by fast Fourier‐transform (FFT) transformation. The low‐magnification TEM image (Fig. 2(a)) of Au@α-Fe_2_O_3_@RGO ternary nanocomposites indicate that the Au and α-Fe_2_O_3_ nanoparticles were dispersed on the RGO sheets with most particles bearing a geometrical spherical structure. Also, fewer larger diameter of Au nanoparticles was found on surfaces of RGO sheets due to aggregation of Au nanoparticles and the individual sheets of the RGO sheets evident in the HRTEM image as shown in Fig. 2(b). The HRTEM image of Fig. 2(c) demonstrates that the dual particles of Au and α-Fe_2_O_3_ nanoparticles were observed on RGO sheets. The Au-nanoparticles exhibits sphere like morphology with the diameter of ~5 to 10 nm, while α-Fe_2_O_3_ nanoparticles measured ~30–40 nm. The HRTEM (Fig. 2(d)) clearly exhibits lattice fringes of Au with the interplanar distance of 0.326 nm, which can be indexed to the (110) plane of the face centers cubic structure of Au. The clear lattice fringes with the interplanar distance of 0.245 nm correspond to (110) plane of the rhombohedral α-Fe_2_O_3_ structure. Moreover, an excellent crystallinity of Au and α-Fe_2_O_3_ on RGO sheets is also confirmed by corresponding FFT transformation as shown in Fig. 2(d). Therefore, we conclude that the Au@α-Fe_2_O_3_@RGO ternary nanocomposites were efficiently synthesized through green reduction of the photochemical method with assisted of chlorophyll.

**Figure 3:**
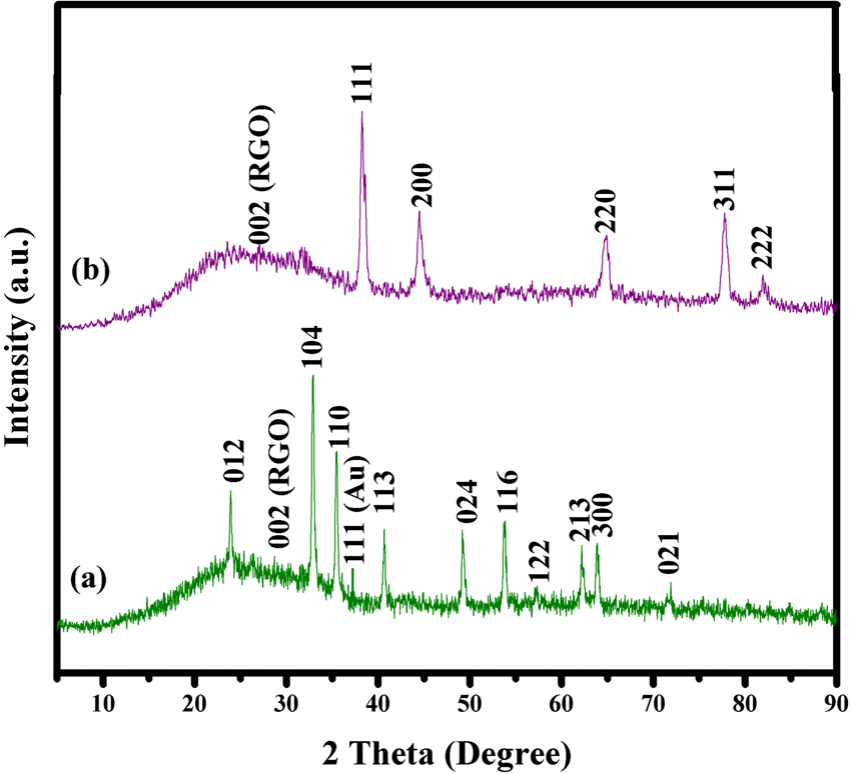
X-ray diffraction analysis of (**a**) Au@RGO and (**b**) photochemically synthesized Au@α-Fe_2_O_3_@RGO nanocomposites.

The structural and phase composition of the Au@RGO and chlorophyll-assisted photochemically synthesized Au@α-Fe_2_O_3_@RGO ternary nanocomposites samples were characterized by powder X-ray diffraction (XRD). Figure 3(a) shows the XRD pattern of Au@RGO nanocomposites and Au with diffraction peaks at 2θ = 38.2°, 44.45°, 65.61° and 77.61°, and 82.61° which can be indexed to (111), (200), (220), (311), and (222) planes of well crystalline Au with cubic phase, along with a broad graphitic peak appears at 23° corresponding to 002 from RGO layers. The crystal sizes of Au nanoparticles estimated to be 8 nm, calculated from Scherer formula. Figure 3(b) shows the XRD pattern of photochemically synthesized Au@α-Fe_2_O_3_@RGO nanocomposites.

The characteristic diffraction peaks of α-Fe_2_O_3_ can be identified at 2θ = 23.97°, 32.78°, 35.56°, 40.71°, 49.17°, 53.77°, 62.29°, 63.92°, and 71.96° which are marked by their Miller indices (002), (104), (110), (113), (024), (116), (122), and (213), (300), and (101), respectively. This diffraction data is consistent with a rhombohedral structure (hematite, JCPDS No. 24-0072) with the lattice parameters of, a = 0.24 nm and c = 0.56 nm. The samples exhibit well crystalline XRD peaks with the average grain sizes of the samples calculated to be 35 nm using Debye–Scherer’s equation. Additionally, the intense peaks at 23° correspond to Miller indices of 002 from RGO layers and low intense peak at 38.2° correspond to 111 phase of Au nanoparticles. The XRD study adds to the evidence that the chlorophyll-assisted photochemical method is suitable for the reduction of Au ions on the surface of Fe_2_O_3_@RGO towards rapid, environment friendly and cost-effective synthesis of ternary nanocomposites.

**Figure 4:**
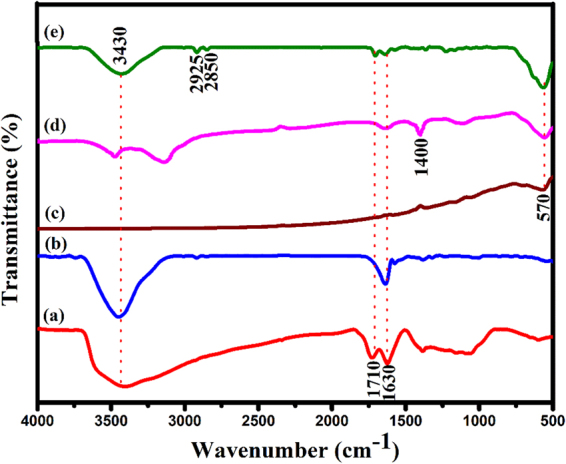
FTIR spectra of (**a**) GO, (**b**) RGO, (**c**) α-Fe_2_O_3_, (**d**) α-Fe_2_O_3_@RGO and (**e**) Au@α-Fe_2_O_3_@RGO ternary nanocomposites.

The functional groups and chemical structures on GO surface, RGO, and Au@α-Fe_2_O_3_@RGO ternary nanocomposites were identified by measuring the FTIR spectra as shown in Fig. 4(a–c). The FTIR spectrum (Fig. 4(a)) of GO shows the broad peak centered at 3436 cm^−1^ corresponding to the stretching vibration of O-H. The weak peak at 1721 cm^−1^ was attributed to C=O stretching of COOH groups^29^. The sharp intense peaks at 1636 and 1044 cm^−1^ corresponds to stretching vibration of C=C and stretching vibration of epoxy (C-O) groups. The FTIR spectrum (Fig. 4(b)) of RGO sheets exhibits new bands at 2928 and 2865 cm^−1^ for the C-H stretch vibrations of the methylene groups and oxygen moieties (C=O and C-O) disappears due to the reduction of GO to RGO under hydrothermal method at 180 °C for 12 h. FTIR spectrum in Fig. 4(c) shows a single and sharp intense peak at 466 cm^−1^ attributed to the stretching vibration of Fe^3+^-O^2−^ bond in the FeO_4_ tetrahedron^7,8,30,31^. The FT-IR investigation of α-Fe_2_O_3_@RGO and Au@α-Fe_2_O_3_@RGO nanocomposite was performed, and the corresponding spectra as shown in Fig. 4(d and e). The FT-IR spectrum of α-Fe_2_O_3_@RGO (Fig. 4(d)) displays four characteristic peaks at 570, 1630, 1400 and 3430 cm^−1^ corresponds to Fe-O vibration in α-Fe_2_O_3_, stretching vibration of C=C, stretching vibration of epoxy (C-O) groups and stretching vibration of water molecules, respectively. The FTIR spectra for the Au@α-Fe_2_O_3_@RGO ternary nanocomposite, Fig. 4(c), exhibits six intense peaks at 570, 1630, 1710, 2850–2925 and 3430 cm^−1^ corresponds to Fe-O vibration in α-Fe_2_O_3_, stretching vibration of C=C, stretching of C=O in COOH groups, C-H stretch vibrations of the methylene groups and stretching vibration of water molecules, respectively^7,8,31,32^. Significance of this studies clearly attributed to the regeneration of electronic holes in the VB of α-Fe_2_O_3_ from excited chlorophyll photoelectrons and there were no observed chemical changes to the α-Fe_2_O_3_ in the ternary nanocomposites following the chlorophyll mediated reduction of Au^3+^ ions.

**Figure 5:**
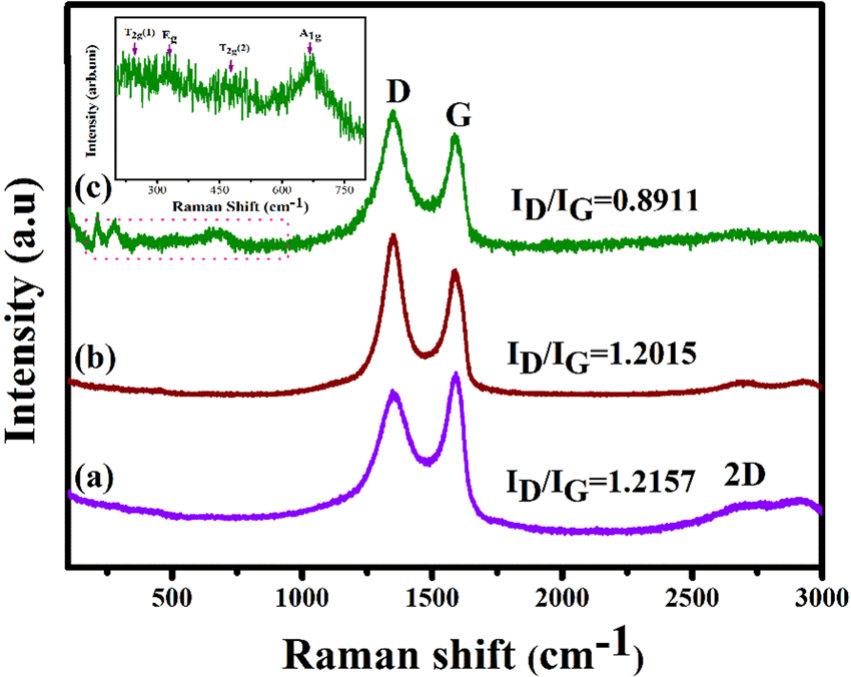
Raman spectra of (**a**) GO, (**b**) RGO and (C) Au@α-Fe_2_O_3_@RGO ternary nanocomposites.

Additionally, Raman spectroscopy was used to characterize the chemical structure of GO, RGO, and photochemically synthesized Au@α-Fe_2_O_3_@RGO ternary nanocomposites. The characterization of the Raman spectra of graphene was helpful in understanding and quantifying defects, phonon, and phonon-electron, electron-electron interaction and identifying number and orientation of graphene layers. Figure 5(a–c) shows the Raman spectra for GO, RGO, and Au@α-Fe_2_O_3_@RGO ternary nanocomposites. The spectra consist of three prominent peaks, namely the D, G and 2D bands. The D band at 1350 cm^−1^ is assigned to the vibrations of sp^3^ carbon atoms of disordered graphite and the G band at 1580 cm^−1^ is associated to the in-plane vibration of sp^2^ (C=C) aromatic carbon structure, which is a particular degenerate phonon mode of E_2 g_ symmetry at the Brillouin zone center. The intense band at 2694 cm^−1^ is associated to the 2D band due to the second-order two phonons with opposite momentum in the highest optical branch adjacent to the K point of the Brillouin zone. An order and disorder crystal structures of graphene have been intensely observed by the intensity ratio (I_D_/I_G_) between D and G band. A universal observation is that the higher disorder graphene leads to a broad D band with higher relative intensity than the G band. The intensity ratio (I_D_/I_G_) of 0.8911, 1.2015 and 1.2157 corresponds to GO, RGO and Au@α-Fe_2_O_3_@RGO nanocomposite, respectively. A broad intense peak at 669 cm^−1^ can be ascribed to the active mode of magnetite A_1g_ and less intense peaks at 540 and 309 cm^−1^ corresponds to the Raman active modes of T_2g_, and E_g_ as shown in the inset Fig. 5(c). The hematite (α-Fe_2_O_3_) has a cubic inverse spinel structure with the Fe^3+^ and Fe^2+^ ions occupying interstitial tetrahedral and octahedral sites and the oxygen ions form an *fcc* closed packed structure symbolized as [Fe^3+^]_-_[Fe^2+^Fe^3+^]O_4_^7,11,29^. The magnetite crystal with the space group of *Fd-3* *m* has five Raman active modes such as A_1g_, three T_2g_, and E_g,_ and four IR active bands (T_1u_). In the centro-symmetrical space group *Fd-3* *m* involves mutual exclusion of IR and Raman vibrational modes. The Fe^3+^ and O^2−^ ions occupy the T_d_ and C_3v_ sites respectively, and contribute to the Raman activity, although bivalent iron cations are not directly involved.

**Figure 6:**
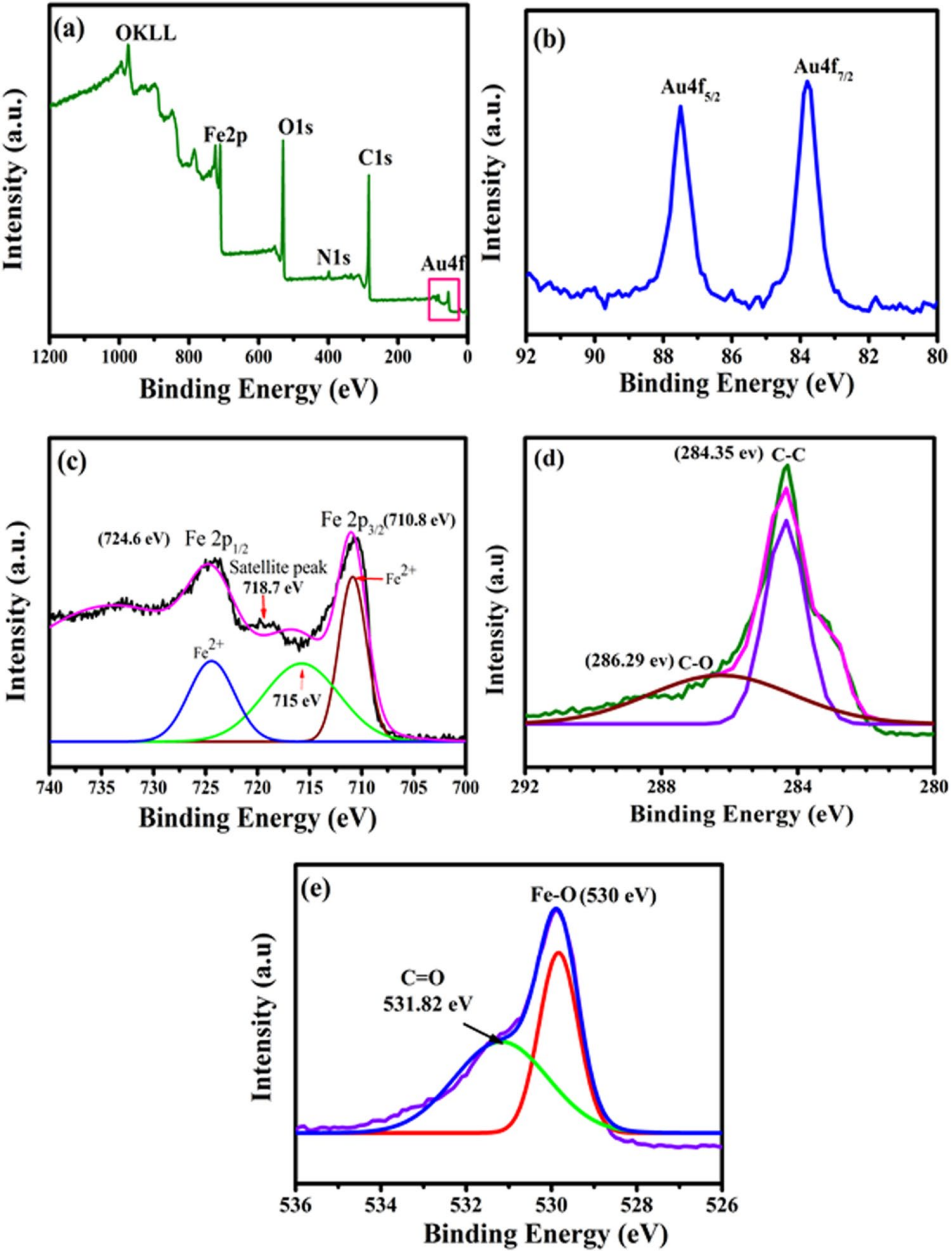
(**a**) XPS survey spectrum of Au@α-Fe_2_O_3_@RGO ternary nanocomposites and high-resolution spectrum of (**b**) Au 4 f, (**c**) Fe 2p, (**d**) C 1s and (**e**) O 1s.

Further, the chemical composition of photochemically synthesized Au@α-Fe_2_O_3_@RGO ternary nanocomposites was determined using X-ray photoelectron spectroscopy (XPS) analysis. The survey spectrum in Fig. 6(a), shows five predominant peaks at 85, 284.35, 530 and 711 eV corresponding to Au 4 f, C 1s, O 1s and Fe 2p, respectively, indicative of Au and α-Fe_2_O_3_ nanoparticles presence on RGO nanosheets surface. Absence of other peaks in the XPS spectrum indicates the high purity of the ternary nanocomposites. Figure 6(b) shows the high-resolution spectrum of Au 4 f with two pronounced peaks at 83.7 and 87.0 eV, associated with Au 4f_7/2_ and Au 4f_5/2_, respectively. This observation is in good agreement with the widely reported XPS spectra of metallic Au° on RGO nanosheets^14,16^. This Au intense peaks indicates that the Au^3+^ ions were completely reduced to Au° metallic nanodots on α-Fe_2_O_3_/RGO ternary nanocomposites under sunlight irradiation with chlorophyll. The high-resolution Fe 2p spectrum in Fig. 6(c) show, five peaks at 710.8, 715, 718.7, and 724.6 eV. The Fe 2p core level is split in to 2p_1/2_ and 2p_3/2_ due to spin-orbit coupling. The photoelectron peaks at 710.8 eV correspond to the binding energies of 2p_3/2_ of Fe^3+^ and Fe^2+^ ions. The peaks at 715 and 710.7 eV can be associated to the 2p_3/2_ of Fe^2+^ and Fe^3+^ respectively. The peak at 725.2 eV corresponds to the binding energy of 2p_1/2_ of Fe^3+^ and Fe^2+^ ions. The observed small peak at 718.7 eV was associated to the satellite peak, revealing the presence of a very small amount of γ-Fe_2_O_3_ or FeO in the nanocomposites. The high-resolution XPS spectrum of C1s characteristic peak for Au@α-Fe_2_O_3_@RGO nanocomposites is shown in Fig. 6(d), which elaborate the contribution from several carbon-oxygen binding arrangements. The C 1s contain non-oxygenated aromatic sp^2^ carbon ring (C-C) at 284.5 eV and oxygenated functional group of carbon sp^2^ C-O at 286.29 eV. The high resolution XPS spectrum of O1s in Fig. 6(e) shows two intense peaks at 530 and 531.82 eV for the anionic oxygen in hematite and residual oxygen functional groups present in RGO sheets. Based on above physico-chemical analytical results, it is clear that the chlorophyll assisted photochemical method is suitable for reduction of noble metal ions to metallic noble nanoparticles on surfaces of graphene based hetero-nanostructures.

### Nucleation-growth mechanism of α-Fe_2_O_3_ and α-Fe_2_O_3_@RGO nanocomposites

**Figure 7:**
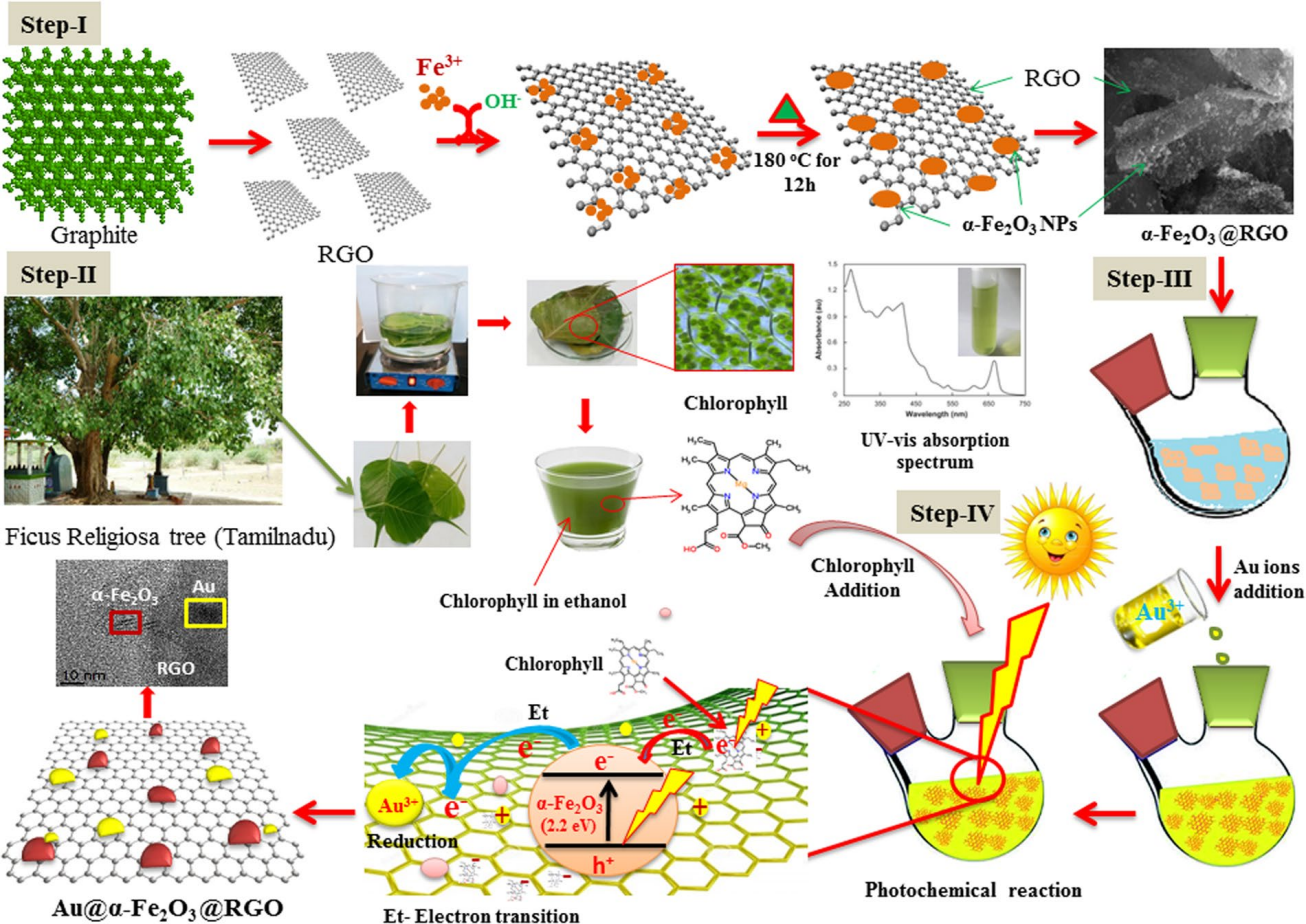
Schematic illustration for the synthesis of α-Fe_2_O_3_@RGO nanocomposites synthesized by hydrothermal process at 180 °C for 12 h and photochemical synthesis of Au@α-Fe_2_O_3_@RGO ternary nanocomposites.

To understand the formation mechanisms of α-Fe_2_O_3_ and α-Fe_2_O_3_@RGO nanocomposite, their nucleation-growth process is best probed based on experimental condition, theoretical investigation, and experimental results as shown in Fig. 7. The simple hydrothermal method was adopted to prepare morphological controlled, high yielding and well crystalline α-Fe_2_O_3_ and α-Fe_2_O_3_@RGO nanocomposite at 180 °C for 12 h. The hydrothermal temperature, solvents, and pH played crucial roles in nucleation-growth mechanism for the formation of well crystalline spherical α-Fe_2_O_3_ nanoparticles. The mechanism is perceived to involve hydrolysis of FeCl_2_.4H_2_O to Fe^2+^ ions and complete formation reaction equations were presented (Equations 1–4). The presence of sodium acetate provides OH^−^ ions, while maintaining pH ≥ 11, resulting in formation of primary FeOOH nucleus with an amorphous structure (reaction Equation 3). The FeOOH monomers are connected by hydrogen bonds until dehydration occurs between the molecules, following the hydrothermal (180 °C) step. Consequently, crystalline α-Fe_2_O_3_ nanospheres are obtained without any further calcination (Equation 4)^23,32^.

1 $$C{H}_{3}CO{O}^{-}+{H}_{2}O\to C{H}_{3}COOH+O{H}^{-}$$

2 $$4F{e}^{2+}+4O{H}^{-}+5{O}_{2}\to 8FeOOH+-2({H}_{2}O)$$

3 $$2{H}_{2}O+4O{H}^{-}+5{O}_{2}+4F{e}^{2+}\to 8FeOOH$$

4 $$2FeOOH\to F{e}_{2}{O}_{3}+{H}_{2}O$$

The synthesis of α-Fe_2_O_3_@RGO is schematically illustrated in Fig. 7 (step-1). In the hydrothermal process, the growth of α-Fe_2_O_3_ nanospheres and reduction of GO into RGO occurred simultaneously, consistent with previous reports that indicate GO can sufficiently be reduced to RGO sheets when exposed to 180 °C for 12 h, without any structural changes^29^. Step.I in Fig. 7 illustrate the entire synthesis of α-Fe_2_O_3_@RGO nanocomposite based on electrostatic-interaction induced self-assembling mechanisms. Following the mixing of FeCl_2_.4H_2_O and GO suspensions, the Fe^2+^ ions are attracted by negatively charged oxygen-containing functional groups (carboxyl, hydroxyl, and epoxy groups) on GO through electrostatic interaction, creating nucleation site^32^. The Fe^2+^ ions are uniformly attached on the GO surface is further hydrolysed by sodium acetate, which releases OH^−^ ions which result in the formation of FeOOH on the GO sheets surface. When the entire mixed solution was transferred to the Teflon-lined stainless steel autoclave and heated at 180 °C for 12 h, the FeOOH phase grows and decomposes to α-Fe_2_O_3_ nanoparticles with sphere-like morphology. Meanwhile, the GO nanosheets are hydrothermally partially reduced into RGO^32^. This method does not use any hazardous reducing agent like ethylenediamine, ammonia, hydrazine hydrate, *etc*.

The complete chemical reactions (Equations 5 and 6) can be expressed as follows:

5 $$2{H}_{2}O+4O{H}^{-}+5{O}_{2}+4F{e}^{2+}/GO\to 8FeOOH/GO$$

6 $$2FeOOH/GO\to F{e}_{2}{O}_{3}/RGO+{H}_{2}O$$

**Figure 8:**
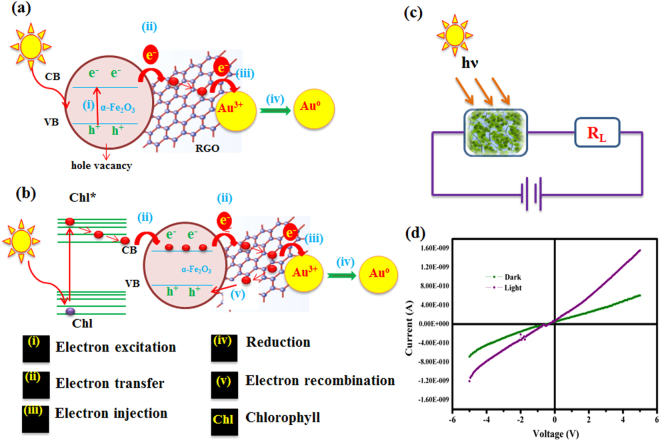
Schematic illustrations of Au^3+^ ions reduction process (**a**) α-Fe_2_O_3_@RGO, (**b**) Chlorophyll-α-Fe_2_O_3_@RGO under visible light irradiation, (**c** and **d**) I-V characteristic of the chlorophyll film.

Step-II in Fig. 7 schematically illustrates the role of α-Fe_2_O_3_@RGO in the photochemical reduction of Au^3+^ ions to Au nanodots on the surfaces of RGO following sunlight exposure to form the α-Fe_2_O_3_@RGO nanohybrids. Generally, Au^3+^ ions were releases when the gold chloride dissolved in ethanol and further this was added into a suspension of α-Fe_2_O_3_@RGO nanohybrids. The Au^3+^ ions were selectively attached with residual functional groups on the surfaces and edges of RGO sheets. Therefore, the Au^3+^ ions and α-Fe_2_O_3_ nanoparticles were dispersed over the surface and edges of the RGO sheets. The α-Fe_2_O_3_ is one of the few binary oxides that have indirect band gap of approximately _˜_2.2 eV that absorb majority visible light from sunlight while still exhibiting excellent aqueous stability. As illustrated in Fig. 8(a), the photoelectrons are excited from valence band to conduction band of α-Fe_2_O_3_ nanoparticles when a quanta of incident light (a photon (hʋ)) with appropriate photon energy is absorbed by the photoactive sites of α-Fe_2_O_3_ in the nanocomposite, producing photoelectrons that migrate on surface of RGO to initiate the reduction of Au^3+^ ions to Au°. The two-dimensional π-conjugation structure of RGO sheets acts as an excellent electron acceptor and transport material, efficiently transferring the electrons for photo reduction of Au^3+^ to Au nanodots formed on the α-Fe_2_O_3_@RGO nanocomposite surface. The RGO hinders the recombination of charge carriers in α-Fe_2_O_3,_ thus increasing the photoelectrons yield. Introduction of chlorophyll chromophores into the reaction causes enhanced absorption of light, results in excited chlorophyll molecules. Generally, the absorbance spectrum of chlorophyll measured through UV-vis absorption spectroscopy in the range of 250 to 750 nm (inset figure in Fig. 7), exhibits two peaks at 646 and 664 nm corresponding to chlorophyll-a and chlorophyll-b, respectively, attesting to strong chlorophyll molar absorptivity in red and blue regions of the visible region, resulting enhanced reduction reaction rate of Au^3+^ ions on the surfaces of α-Fe_2_O_3_@RGO nanocomposite. The detailed investigation was presented in below. As shown in Fig. 8(b) and delineated in Equations 6–9 explains the detailed reduction mechanism Au^3+^ ions on α-Fe_2_O_3_@RGO nanocomposite under visible light irradiation with assisted of chlorophyll. With the presence of visible light, the chlorophyll gets excited and it was generated photoelectrons. These photoelectrons are transferred to conduction band of α-Fe_2_O_3_ nanoparticles, and then it was migrating on surface of RGO to initiate the reduction of Au^3+^ ions. In this reaction process more photo-induced electrons contribute to reduction of Au^3+^ ions to Au° metallic nanodots with very rapid time. Meanwhile, the electronic vacancy in the VB holes of α-Fe_2_O_3_ was regenerated through the excess of photoelectron transfer to prevent the structural loses of α-Fe_2_O_3_. The following chemical reactions (Equations 7–10) expressed the formation of ternary nanocomposites using chlorophyll^28,32,33^.

7 $$\alpha -F{e}_{2}{O}_{3}/RGO+h\nu \to \alpha -F{e}_{2}{O}_{3}(h+e)/RGO$$

8 $$\alpha -F{e}_{2}{O}_{3}(3e)/RGO+A{u}^{3+}\to \alpha -F{e}_{2}{O}_{3}/RGO+Au$$

9 $$Chl+h\nu \to Ch{l}^{\ast }(e)$$

10 $$\alpha -F{e}_{2}{O}_{3}(2h)/RGO+Ch{l}^{\ast }(2e)\to \alpha -F{e}_{2}{O}_{3}(e)/RGO+Chl$$

Moreover, the following studies could be supported for the formation of Au@α-Fe_2_O_3_@RGO nanocomposite. The proposed formation mechanism of chlorophyll (concentration- 2 g leaves in 5 mL of ethanol) was coated on an ITO glass substrate by drop casting method and its electrical response under visible (mercury lamp) light illumination was evaluated for current-voltage (I-V, Keithley 2400 Sourcemeter) as shown in Fig. 8(c and d). The I-V chlorophyll film response shows the rectification behavior (diode characteristics)^34^. In the darkness, chlorophyll film acts as a diode in reverse bias, where virtually no current flows even with increased voltage. When the chlorophyll film was exposed to visible light, the current increased rapidly, with resultant 10 times higher current compared to response in absence of light illumination Fig. 8(d). Based on the I-V characteristic curve it was also evident that the resistance value of the chlorophyll film decreased when irradiated with visible light, attesting to excitation of chlorophyll and the emanating photoelectrons, which are transported to the conductive RGO sheets, triggering a rapid reduction of Au^3+^ ions to metallic Au° clusters on the surface of nanocomposite, and potentially regenerating the electronic hole vacancy in the VB of α-Fe_2_O_3_.

**Figure 9:**
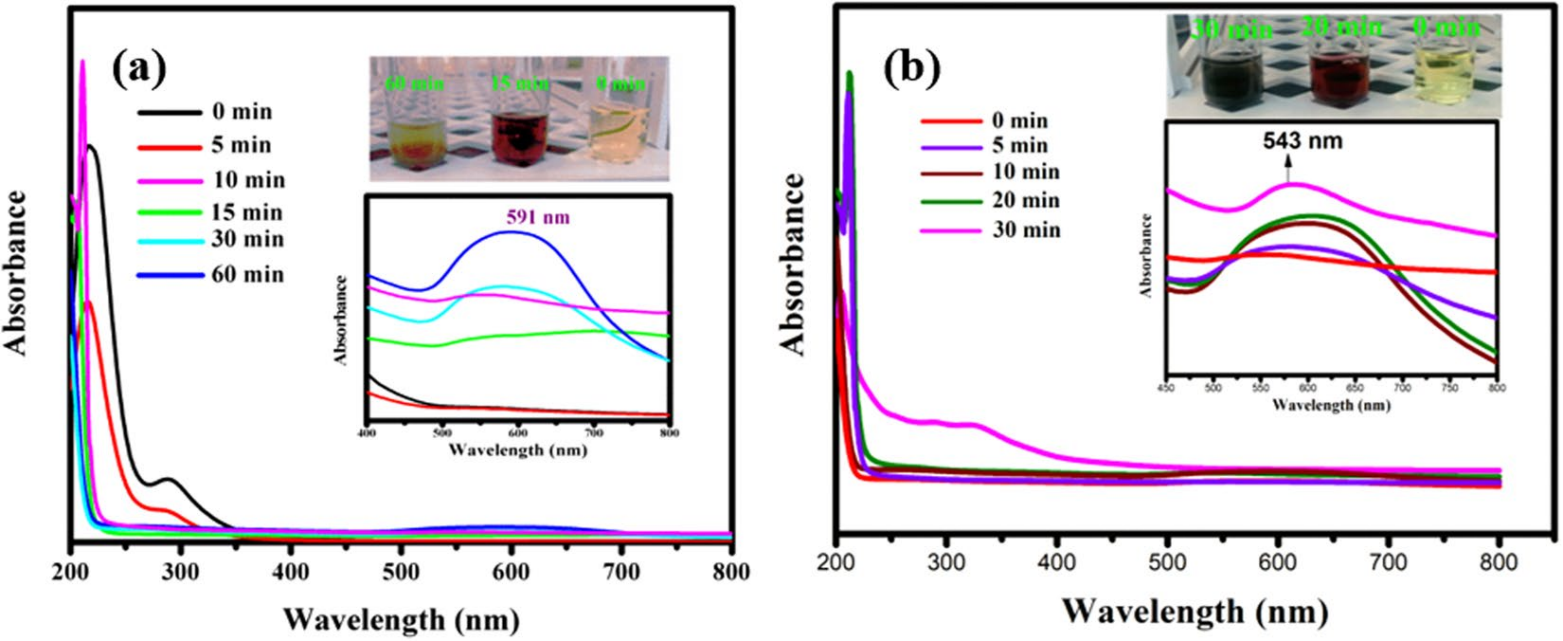
UV-Vis absorption spectra (**a**) Au^3+^ ions reduction on α-Fe_2_O_3_@RGO under visible light irradiation (0 to 60 min) without chlorophyll and (**b**) Au^3+^ ions reduction on α-Fe_2_O_3_@RGO with chlorophyll under visible light irradiation (0 to 30 min).

To further explore the role of chlorophyll in the photochemical reduction process, the UV-vis absorption spectrum was used to monitor the synthetic process. Figure 9(a and b) exhibits the optical effects of reduction of Au^3+^ ions on surfaces of α-Fe_2_O_3_@RGO under visible light irradiation with and without chlorophyll. The suspension color changes from yellow to red during the first 15 min of visible light irradiation, with an evident absorption peak at ~500 nm (Fig. 9(a)), suggesting a reduction of Au^3+^ ions to Au° metallic nanodots. After 60 min irradiated suspension shows dark red color with a corresponding absorption peak at ~591 nm (Fig. 9(a)), indicative of the complete reduction of Au^3+^ to Au nanodots on the α-Fe_2_O_3_@RGO nanosheets surfaces. In contrast, the addition of chlorophyll to the reaction, the results in color change from yellow to dark red within 30 min with a correspondent absorption peak at ~543 nm (Fig. 9(b)). This phenomenon attests to the rapid chlorophyll mediated catalysis of the reduction of Au^3+^ to Au° metallic nanodots under visible light irradiation. Notably, chlorophyll not only drastically reduces the reaction time, but the intense absorption peak ~543 nm, is an evidence of formation of smaller Au nanodots compared to synthesis carried out in absence of chlorophyll. The phenomenon is consistent with the well-known observation that as the reduction reaction time increases the size of the critical nuclei gradually increases, and a correspondent red shift (500 to 591 nm) occurs due to the enlargement of the Au nanodots^33,35–39^. As such, it is convincing that that the chlorophyll induces the rapid rate of reduction and concomitantly prevents the aggregation of Au, resulting in ultra-small nanodots of Au grown on surfaces of α-Fe_2_O_3_@RGO nanosheets.

### Enhanced photocatalytic activity

**Figure 10:**
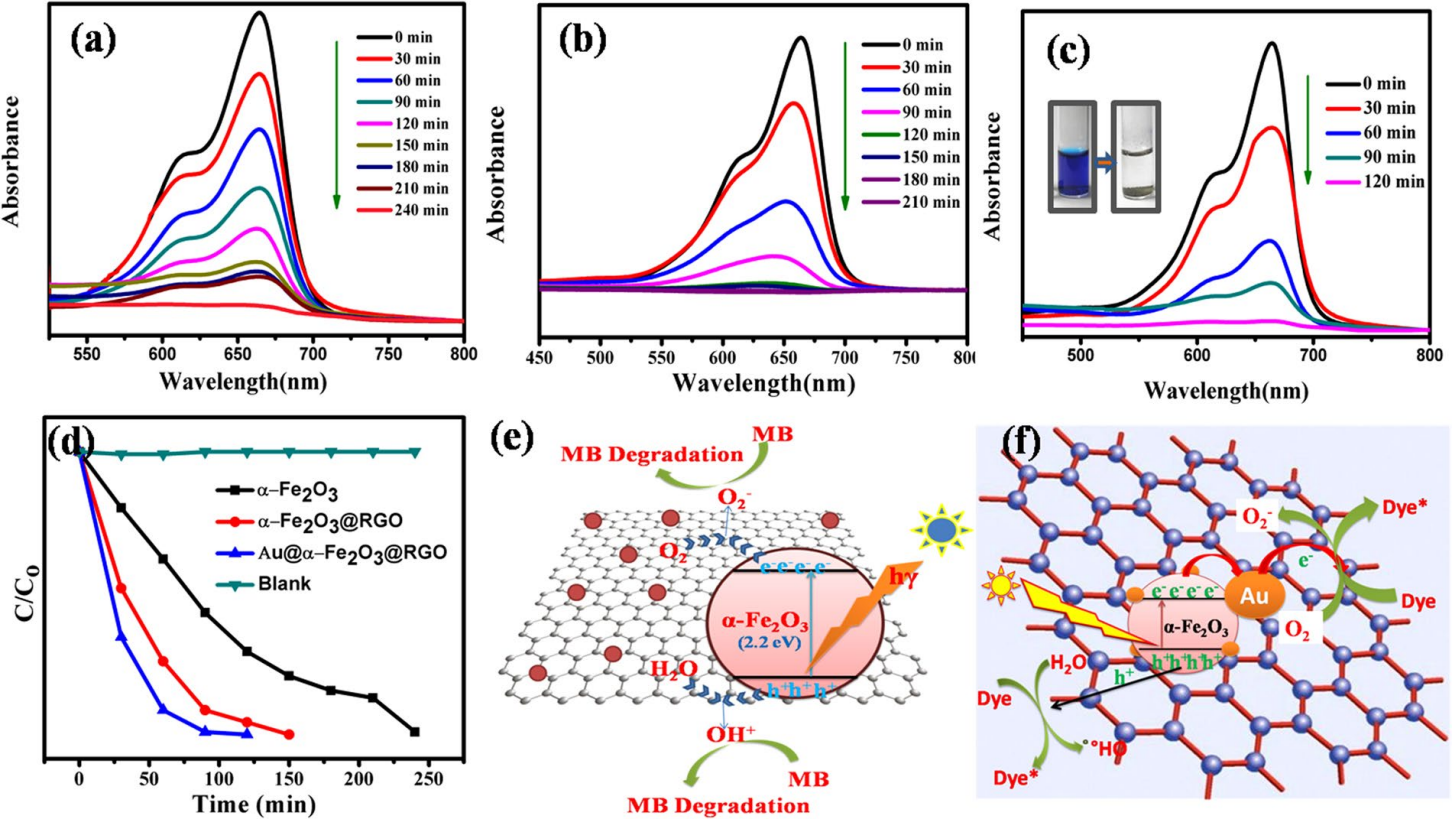
UV spectra of methylene blue dye degradation using α-Fe_2_O_3_ (**a**), α-Fe_2_O_3_@RGO (**b**), Au@α-Fe_2_O_3_@RGO nanocomposites (**c**), and degradation rate (C/C_o_) (**d**) photocatalyst under visible light and photocatalytic degradation mechanism of α-Fe_2_O_3_@RGO (**e**), Au@α-Fe_2_O_3_@RGO (**f**) nanocomposites.

The Au@α-Fe_2_O_3_@RGO ternary nanocomposite has the potential to work as efficiently, multifunctional materials for photocatalytic and energy conversion harvesting applications. This nanocomposite may exhibit better photocatalytic properties by the improvement of their electronic and structural properties than pure photocatalysts. Among all the recent photocatalytic materials, the Au@α-Fe_2_O_3_@RGO nanocomposite is a unique advantage in suppressing the charge recombination and faster electron transfer due to higher conductivity, chemically stable and favorable band gap (2.1–2.2 eV) to absorb photons in the visible light range. The photocatalytic activity of α-Fe_2_O_3_, α-Fe_2_O_3_@RGO, and Au@α-Fe_2_O_3_@RGO nanocomposites was evaluated by following the decomposition of the methylene blue (MB) dye in water under visible light irradiation at various time intervals by measuring the absorbance spectra using a UV-visible spectrophotometer. Figure 1(a–c) shows the optical absorbance spectra at 450–800 nm, for the photocatalytic system resulting from the photochemical degradation of this dye for all the photocatalyst samples. To evaluate the photocatalytic performance of the as-prepared photocatalysts, the UV-Vis absorption spectra of the illuminated samples at different time intervals were observed as shown in Fig. 10(a–c). It can be seen from Fig. 10, that methylene blue (MB) was degraded under visible light irradiation in the presence of different photocatalysts and degradation was not observed in the dark, even in the presence of photocatalyst. The maximum absorbance peak for methylene blue is observed at 664 nm and the photocataytic results was collected from 0 min till the discolorization of dye occurs at a regular interval of 30 minutes. The MB dye was fully degraded within 240, 180 and 120 min corresponds to the photocatalysts of α-Fe_2_O_3_, α-Fe_2_O_3_@RGO, and Au@α-Fe_2_O_3_@RGO nanocomposites, respectively as shown in Fig. 10(a–c). Figure 10(d) represents the graph of MB dye degradation rate. The plotted graph is obtained by irradiation time versus log of C/C_o_ value. The Au@α-Fe_2_O_3_@RGO nanocomposite exhibits higher degradation rate than that of as-prepared α-Fe_2_O_3_ and α-Fe_2_O_3_@RGO nanocomposites. Moreover, a control experiment (blank) showed that the degradation was not attained due to the absence of photocatalysts.

**Figure 11:**
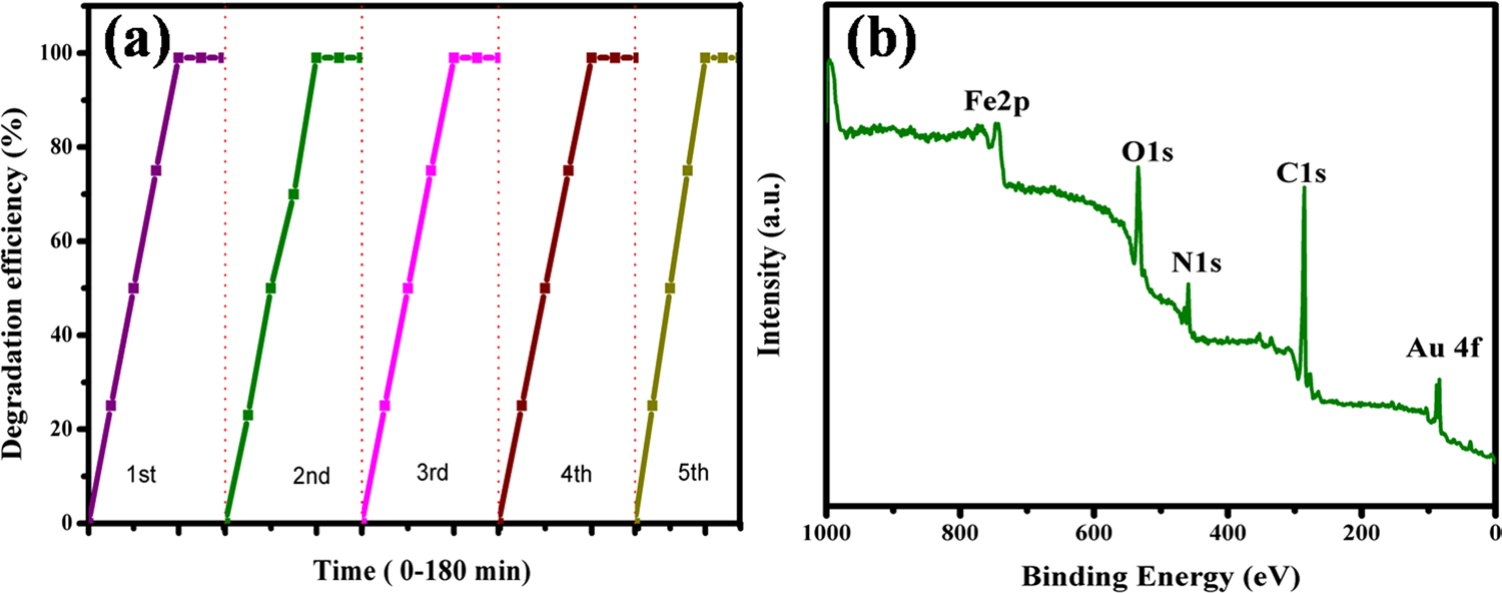
(**a**) Recyclability of Au@α-Fe_2_O_3_@RGO nanocomposites in the degradation of methylene blue and (**b**) XPS analysis of Au@α-Fe_2_O_3_@RGO nanocomposites and this sample was collected from after 5^th^ cycle.

Moreover, photocatalytic mechanism of α-Fe_2_O_3_, α-Fe_2_O_3_@RGO, and Au@α-Fe_2_O_3_@RGO nanocomposites were efficiently described to understanding the photocatalytic behavior of as-prepared photocatalysts. Figure 10(e and f) demonstrates the photocatalytic mechanisms of α-Fe_2_O_3_@RGO, and Au@α-Fe_2_O_3_@RGO nanocomposites. Basically, the α-Fe_2_O_3_ and graphene-based nanocomposites have the potential to work as efficiently, multifunctional materials for energy conversion & storage applications. These composites may exhibit better photocatalytic properties by the improvement of their electronic and structural properties than pure photocatalysts of α-Fe_2_O_3_. Generally, the α-Fe_2_O_3_ nanoparticles are widely used as high-performance photocatalysts because of it’s cost-effective, chemically stable and favorable visible range band gap (2.1–2.2 eV) to absorb photons in the visible light range. However, its photocatalytic efficiency is delayed by high charge recombination rate causes to decrease the photodegradation efficiency against methylene blue. The complete degradation of methylene blue was achieved at 240 min (Fig. 10(a)) when used α-Fe_2_O_3_ NPs as a photocatalyst. The lower photodegradation efficiency of α-Fe_2_O_3_ NPs may due to the high charge recombination rate because of boundaries between the particles. In α-Fe_2_O_3/_RGO nanohybrid materials, α-Fe_2_O_3_ acts as a photocatalyst, to excite the electrons from the valence band (CB) to the conduction band (CB) under visible light range and create electron (e^−^) and hole (h^+^) pairs, which can migrate into RGO and initiate photocatalytic reactions as shown in Fig. 10(e). The holes are trapped by the H_2_O to form active hydroxyl radicals (OH) and superoxides (O_2_˙, HO_2_˙). These ˙OH and superoxide (O_2_˙, HO_2_˙) are strong oxidant species could be degrading organic dye molecules. Graphene acts as an excellent electron-transport material in the process of photocatalysi, so that the hybridization of α-Fe_2_O_3_ with RGO can hinder the recombination of charge carriers and increase the photoelectrocatalytic performance than α-Fe_2_O_3_ NPs. The electronic interaction between α-Fe_2_O_3_ NPs and RGO sheets was a vital factor, which could increase the separation of photogenerated electrons-holes (e^−^h^+^) pair significantly, resulting in an increase in the number of electrons/holes participating in the photodegradation process. To further enhance the photocatalytic performance by using photocatalysts, the plasmonic Au as a co-catalyst was loaded onto the α-Fe_2_O_3_/RGO nanocomposites and the inclusion of the Au nanoparticles on the α-Fe_2_O_3_/RGO served to increase the overall light absorption of the photocatalysts. The detailed photocatalytic mechanism was presented in Fig. 10(f). Under visible-light irradiation, electrons and holes are generated in the conduction and valence bands, respectively. Co-catalysts such as plasmonic Au and RGO may improve the charge separation and suppress the recombination of excited photogenerated carriers. The suppressed charge recombination and enhanced charge separation in α-Fe_2_O_3_ is due to extraction of the photogenerated electrons from α-Fe_2_O_3_ to Au and RGO, which resulted in the enhanced photocatalytic activity toward degradation of methylene blue within 120 min under visible light irradiation as shown in Fig. 10(c). After completing the photocatalytic reaction, recycling of the Au@α-Fe_2_O_3_@RGO nanocomposites is the most vital parameter to determine the sustainability of this Au@α-Fe_2_O_3_@RGO photocatalyst. The recyclability experiment was performed to estimate the efficiency of the Au@α-Fe_2_O_3_@RGO photocatalyst and we found that the Au@α-Fe_2_O_3_@RGO photocatalyst can be used repeatedly to carry out photodegradation of various dye molecules. As shown in Fig. 11(a), the degradation efficiency of methylene blue decreased very slightly after five consecutive reaction cycles. In addition, the XPS analysis verified that its chemical structure remained unaffected after the photocatalytic reaction as shown in Fig. 11(b). Therefore, the Au@α-Fe_2_O_3_@RGO nanocomposites were stable, efficient, and non-photocorrosive, which are significant, features for practical photocatalytic applications.

## Conclusions

In summary, we have prepared a chlorophyll mediated Au@α-Fe_2_O_3_@RGO ternary nanocomposites through photochemical method under sunlight irradiation. The detailed photochemical reduction mechanisms of Au^3+^ ions on α-Fe_2_O_3_@RGO nanocomposites were demonstrated. An excited state of chlorophyll generates more photoelectrons and it does contribute to reduction of Au^3+^ ions to Au° metallic nanodots on surfaces of α-Fe_2_O_3_@RGO hetero-nanostructure. This study demonstrates that the chlorophyll induces the rate of reduction reaction and prevents the aggregation of Au, resulting in Au nanoparticles formed on α-Fe_2_O_3_@RGO surface, with a rapid synthesis time of 30 min. The structural, chemical and optical analysis of as-prepared samples were characterized through XRD, FTIR, Raman spectroscopy, UV-Vis spectroscopy and XPS analysis. The described method demonstrates a cost-effective, rapid, surfactant less, green reduction and scalable method for preparation of ternary nanocomposites. Moreover, the photochemically synthesized Au@α-Fe_2_O_3_@RGO ternary nanocomposites were used as high performance catalyst toward degradation of methylene blue under visible light irradiation. The suppressed charge recombination and enhanced charge separation in α-Fe_2_O_3_ is due to extraction of the photogenerated electrons from α-Fe_2_O_3_ to Au and RGO, which resulted in the enhanced photocatalytic activity toward methylene blue degradation with high chemical stability. This present study may pave a way for improving the photoelectrocatalytic activity to produce large scale evolution of H_2_.

## Experimental Methods

### Materials

All chemicals were of analytical grade and used as received without further purification. Graphite flakes (~105 µm flakes), potassium permanganate (KMnO_4_), aspartic acid, sodium nitrate (NaNO_3_), gold(III) chloride trihydrate (HAuCl_4_._3_H_2_O) Sodium hydroxide (NaOH), Ferrous chloride tetrahydrate (FeCl_2_·4H_2_O) were supplied by Sigma Aldrich.

### Synthesis of graphene oxide

Graphene oxide (GO) was synthesized through modified Hummer’s method. Briefly, 2 g of preheated natural graphite flakes and 4 g of NaNO_3_ were added to 100 ml of concentrated H_2_SO_4_ and stirred in an ice bath. Subsequently, 4 g of KMnO_4_ was gradually added to the above mixture and it was kept at below 10 °C for 6 h. This solution was further stirred at room temperature for 4 h. To the resulting brownish paste, 100 ml of distilled water was added, and the suspension stirred for 2 h. This was followed by a slow addition of 10 ml of 33% H_2_O_2_, resulting in a yellow suspension Finally, the precipitate was filtered and washed with 5 ml of HCl (5%) followed copious washing with deionized water, to neutral pH. The obtained product was dried under vacuum at 70 °C for overnight.

### Synthesis of α-Fe_2_O_3_@RGO nanocomposite through hydrothermal method

The α-Fe_2_O_3_@RGO nanocomposites were prepared through the hydrothermal method. Briefly, 20 mM of FeCl_2._4H_2_O and 40 mM of sodium acetate were dissolved in 30 ml of GO suspension with constant stirring for 10 min. The suspension was sealed in a 75 mL Teflon lined stainless steel autoclave and placed in an oven and heated at 180 °C for 12 h. After cooling to room temperature, the obtained precipitate was collected and copiously washed with deionized water followed by ethanol. The product was dried overnight in vacuum oven at 70 °C. Pristine α-Fe_2_O_3_ nanoparticles were also synthesized by similar hydrothermal preparation process in the absence of GO solutions.

### Chlorophyll-assisted photochemical synthesis of Au@α-Fe_2_O_3_@RGO ternary nanocomposite

A100 mg of α-Fe_2_O_3_/RGO nanocomposites was dispersed in 20 ml of 99.9% ethanol. This was followed by addition of 50 mM of HAuCl_4_._3_H_2_O dissolved in 10 ml of ethanol. Subsequently, 5 ml of chlorophyll in ethanol suspension was added. The chlorophyll used had been extracted with 99.9% ethanol from 2 g of *Ficusreligiosa* leaves collected near Salem-Tamilnadu, India, and its chromophore characteristics studied by UV-VIS absorption spectroscopy in the range of 250 to 750 nm. The mixed solutions were placed in sunlight and 2 ml aliquot was withdrawn every 5 min for UV-Vis analysis. The color of suspension changes from bright yellow to red under sunlight irradiation. After 20 min irradiation, the suspension was collected and centrifuged for 30 min. The final product was dried overnight under vacuum oven at 70 °C. Similar photochemical synthesis process was conducted for synthesis of Au@α-Fe_2_O_3_@RGO ternary nanocomposite in absence of chlorophyll.

### Characterization

The XRD measurements were carried out at room temperature using a PAN analytical (X-Pert-Pro) diffractometer with a Cu Kα_1_ radiation (λ = 1.5406 Å) over a scanning interval (2θ) from 10 to 90°. The average crystallite sizes were estimated using the Scherrer formula. The average crystallite sizes were estimated using the Scherrer formula. The morphology and elemental analysis of the composites were determined by HRTEM and FESEM (FEI Quanta – 250) with EDX and elemental mapping. The infrared spectrum of the samples was obtained by using a FTIR spectrometer (Bruker Tensor 27, Germany), with samples prepared by a KBr pellet and spectra ranging from 4000 to 450 cm^−1^ acquired. Raman scattering was performed on a JY-1058 Raman spectrometer using a 520 nm laser source. UV-Visible spectral analysis was done by JoscoV-650 spectrophotometer. The X-ray photoelectron spectroscopy (XPS) spectra were obtained on a Kratos Axis Ultra-DLD X-ray photoelectron spectroscope (Manchester, U.K). Surface area and pore size distribution of a nanocomposite was determined using a micromeritics ASAP 2020 surface area analyzer.

### Photocatalytic degradation of methylene blue under visible light

The photocatalytic activity of α-Fe_2_O_3_, α-Fe_2_O_3_@RGO and Au@α-Fe_2_O_3_@RGO nanocomposites were studied through methylene blue used as a modal dye to determine their photocatalytic activity under visible light irradiation. In detail, 0.0372 g of Methylene blue (MB) was dissolved in 100.0 ml of doubly distilled water so that the concentration of dye solution was 1.0 × 10^−3^ M. It was used as a stock solution. This solution was further diluted to 10 ppm concentration for these experiments. The optical density of this dye solution was determined with the help of a UV spectrophotometer. The 5 mg of the catalysts were taken and dispersed in 50 ml of aqueous solution of methylene blue. The solution was mixed by using a stirrer constantly in the presence of dark environment for 15 minutes and then irradiated in the visible sun light. During the exposure of dye solution to the visible sunlight source, aliquots were taken out at intervals of every 30 minutes and the concentrations of methylene blue were determined by UV-Vis Spectrometer. The absorption spectrum is obtained from UV-visible spectrometer by a plot between absorbance versus irradiation time and the efficiency of decolorization is determined in term of change in intensity of absorption maximum of the dye with respect to time they are exposed to sunlight. The efficiency of decolorization is calculated using the formula

11 $${{\rm{C}}}_{0}-{\rm{C}}/{{\rm{C}}}_{0}\ast 100 \% $$

In addition to that, the apparent rate constant of photocatalytic degradation of Methylene blue (MB) is calculated using the expression

12 $${\rm{In}}\,{\rm{C}}/{{\rm{C}}}_{0}=-{\rm{kt}}$$

Where, **C**_**0**_ – Initial concentration of dye solution, **C** – Concentration of dye after irradiation, **K** – Absorption rate constant of the reaction and **t** – time required to complete degradation.

### Data availability statement

The authors declare that the data is available, and can be provided at any stage.
